# Analysis of the Human Scent on Fired Cartridge Cases from a Simulated Crime Scene

**DOI:** 10.1021/acs.analchem.4c06231

**Published:** 2025-02-14

**Authors:** Ulrika Malá, Václav Vokálek, Pavel Vrbka, Jana Čechová, Petra Pojmanová, Oleksii Kaminskyi, Veronika Škeříková, Štěpán Urban

**Affiliations:** † University of Chemistry and Technology in Prague, Faculty of Chemical Engineering, Department of Analytical Chemistry, Technická 5, 160 00 Prague, Czech Republic; ‡ Regional Group for Cynology and Hippology, Odorology Section for Brno, Police of the Czech Republic, U Dálnice 1, 664 42 Modřice, Czech Republic; § University of Chemistry and Technology in Prague, Faculty of Chemical Engineering, Department of Physical Chemistry, Technická 5 160 00, Prague, Czech Republic

## Abstract

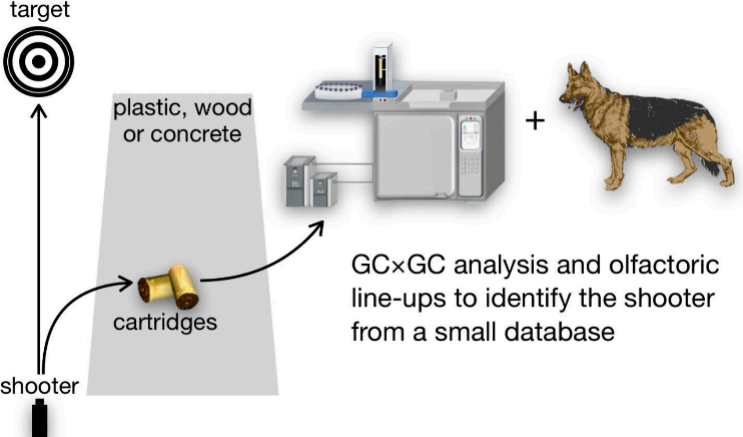

Fired cartridge cases are often found at crime scenes connected with a shooting, and their prompt analysis can be very useful for the police investigation. In addition to dactyloscopy (fingerprints) that tends to be more or less damaged on the cartridges and often are not adequate for individual identification, there are also scent traces on the fired cartridges that are not fully destroyed by the gun’s being fired. In this pilot study, we compare the human scent remaining on cartridge cases after firing with scent samples from different volunteers to find out who loaded the gun before the gun was shot. In this experiment, a simulated crime scene was prepared, and one of our volunteers loaded the weapon. Analysis of the scent remaining on cartridge cases was carried out using two different methods, namely, olfactronics and olfactorics.

## Introduction

Forensic olfactronics is a new field of forensic analysis whose aim is to analyze scent samples using advanced analytical chemistry techniques.^1−3^ Unlike olfactorics (the odorology method using specially trained canines), forensic olfactronics is an objective method. On the other hand, the olfactory system of a canine is still more sensitive than the best contemporary instruments of analytical chemistry usable for human scent analysis. This is why both methods should be used for these purposes in the future during criminal investigations, so they can complement each other and be a desirable chain of evidence to present before the court. Cartridge cases are often one of the traces that can appear on the crime scenes usually connected with a shooting, mainly in the cases of very serious crimes. Unfortunately, the fingerprints tend to be partially spoiled; thus, they cannot be compared with the dactyloscopy database. In a previous study,^4^ it was found that the less volatile substances that seem to be more significant for the individual identification of human scent^5^ resist high temperatures for a short time (up to 500 °C for 1 min). That led to a presumption that at least a part of the human scent remaining on the cartridge after loading the gun and even after the gun’s firing would be sufficient for an individual identification.^4^

## Experimental Section

### Preparation of the Samples

First, the glass beads used as a sorbent of human scent were cleaned in chromosulfuric acid (prepared at the UCT Prague) and washed in deionized water, ethanol (for UV–VIS spectroscopy, min 99.8%, Penta, CZ), and hexane (quality for GC-MS, Sigma-Aldrich, USA). After being dried (270 °C, 90 min), the glass beads were stored in a desiccator.

During the preparation of the sorbents before sampling, the cartridges were carefully cleaned before the scent itself was collected on it. Despite neither the cartridge cases nor the ammunition being prewashed at the crime scene, it was thought at the beginning of the study that the lubricants used for manufacturing ammunition could be problematic during the data evaluation. In this case, the cartridges were cleaned before sample collection to have an optimal analytical background. The cartridges were prewashed before sampling in a mixture of ethanol and hexane in a ratio of 7:3 for 2 × 20 min. A new mixture was used for the next 20 min of bathing. The measurement results of the samples collected on prewashed cartridges and the cartridges only taken out of the original package were compared.

Based on a certified and published methodology of scent collection,^6^ the sampling procedure was carried out on both sorbents: glass beads as well as cartridges. The sampled glass beads were then extracted into ethanol and concentrated to 70 μL, based on a previous study.^7^ For the olfactoric lineups, the commercial fabric Aratex (70% cotton, 25% viscose, 5% polyester, 280 g/m2, purchased from CHLUM-TEX, CZ) was used.

Our simulated crime scene with a heterogeneous nature was created in the basement with brick walls and a concrete floor. On the floor, there was a wooden palette and plastic sheet on it. The cartridge cases from our simulated crime scene were collected 15 min after the gun was fired and then compared with volunteer samples using two different methods: the olfactronics using the two-dimensional gas chromatography with mass spectrometry and the olfactoric method using specially trained police canines. For the first method, 12 samples from four different volunteers were sampled and compared with the scent obtained from the cartridge cases to determine which of the volunteers had loaded the gun. Two of our four unknown samples (cartridge cases with the same scent) were collected from the plastic sheet, one from the wooden palette, and one from the concrete floor.

### Instrumentation

For this experiment, only one gun type was used (a Sauer 38H, Sauer Sohn Germany, caliber 7.65 Browning, ammunition, type FMJ 73 grs, produced by Sellier Bellot, Czech Republic).

The samples were measured by a 7980B GC two-dimensional gas chromatograph (Agilent, USA) coupled with a Pegasus BT 4D time-of-flight analyzer mass spectrometer (LECO, USA). The columns were connected in reverse order. The primary column was a semipolar Rtx-200MS (30 m + 2 m precolumn, Restek, USA), and the secondary column was a nonpolar TG5-HT (1.1 m, Thermo Fisher Scientific, USA). The diameter of the columns was 0.25 mm, and the thickness of the stationary phase was 0.25 μm. Helium (purity 5.5, Linde CZ) was used as a carrier gas for gas chromatography. The sample was injected in a volume of 1 μL in splitless mode (2 min) at a temperature of 280 °C. The temperature gradient on the primary column started at 40 °C, held for 2 min, and ended at 320 °C, held for 10 min. The temperature increased at a rate of 5 °C/min. The temperature on the secondary column was always 5 °C higher than that on the primary column. The modulator between the two columns always had a temperature 15 °C higher than the secondary column. The cryogenic modulation at −80 °C with three modulation periods of 6, 8, and 10 s were used. The carrier gas flow rate was 1.5 mL/min. The interface between GC and MS was heated to 280 °C. The mass detector used electron ionization with an energy of 70 eV ionizing electrons, and the temperature of the ion source was 250 °C. Data collection took place in TIC (total ion current) mode, with a mass interval of 29–800 (*m*/*z*); data collection took place at a speed of 200 spectra/s. The time required to elute the solvent was set to 500 s. To increase the detection sensitivity, a 200 V higher voltage was set on the detector compared to tuning. The ratio of the signal and the noise was set at S/N 300.

### Evaluation of the Data

The measured chromatographic data were first aligned by reference chromatographic peaks that occurred in all the scent samples. These peaks were used as anchorage points for aligning all of the peaks from different chromatograms. After the data alignment, only the substances that occurred at least in one of four “unknown” cartridge cases were used. This approach was chosen to focus our analysis on substances that come from the human scent and not a background of the sorbent itself, contaminants, etc. Additionally, only substances that occur at least in 75% of all of the samples from the volunteers were chosen. However, the substances themselves are not significant for individual identification; what is important are the ratios of the peak areas. All of the area ratios were then compared with all of the analyzed samples and ordered by their increasing statistical variance. Hence, only those that are stable over time can be chosen and are more probably behind the genetically determined part of the human scent. Just the first 150 of the area ratios for the statistical evaluation were chosen. The results were statistically evaluated by the preview method, cluster analysis.

### Olfactoric Lineups

The same set of unknown samples from the simulated crime scene was used for comparison with 19 different volunteers by specially trained police canines. Their results are normally used in police practice as nondirect evidence before court. The lineups were carried out in special rooms designed for odorology comparisons. Every lineup comparison was double-blind. A special fabric (Aratex), applied in the Czech Republic as a scent sorbent for police odorology, which was exposed to the cartridge case for at least 30 min (30 min, 1 h, 6 h, and 1 day), was used as sniffing samples. The cartridge cases were stored in a glass jar on the sorbent for a defined period. This fabric, used as a secondary sorbent for scent samples, was employed due to the canines’ training. In each lineup test, there were five volunteer samples, and the canine compared them with the sample from the simulated crime scene. When it finds the match, it marks the jar with the specific sample by laying down at it. The random interest control was performed before every comparison.

## Results and Discussion

**Figure 1:**
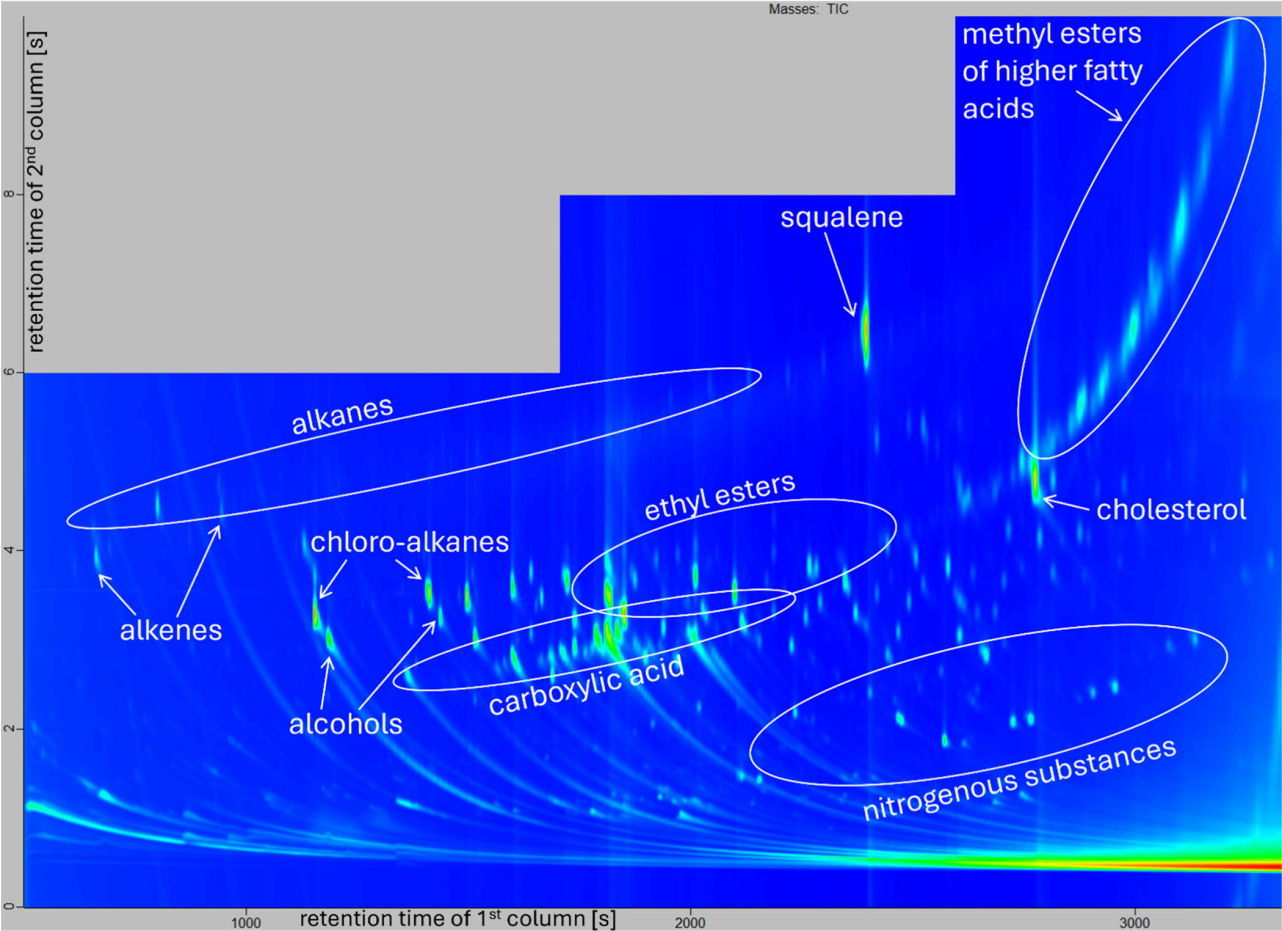
Chromatogram of a volunteer’s sample from our small database captured on the glass beads. The *x* axis represents the retention time of the first column [s], and the *y* axis represents the retention time of the second column [s]. The areas corresponding to individual groups of substances are indicated in the image.

**Figure 2:**
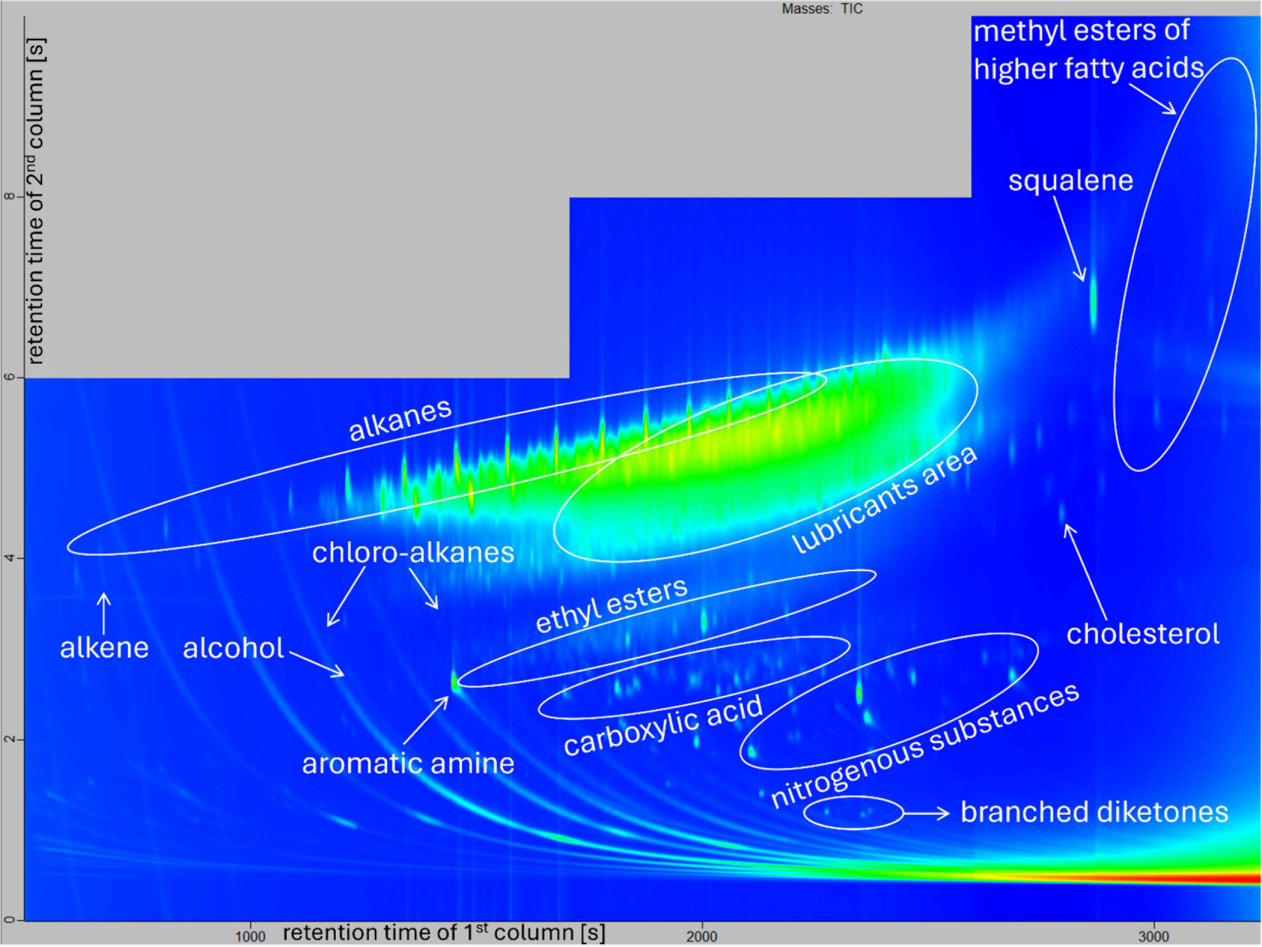
Chromatogram of an unknown sample from the cartridge case. The *x* axis represents the retention time of the first column [s]; the *y* axis represents the retention time of the second column [s]. The areas corresponding to individual groups of substances are indicated in the image.

**Figure 3:**
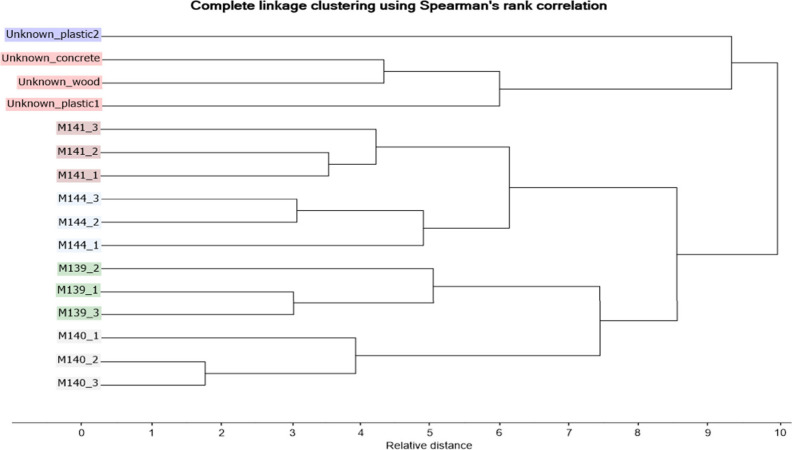
Cluster analysis of the scent samples from the volunteers and the unknown samples from the cartridge cases collected on the simulated crime scene from different surfaces. M refers to the male sample. The first number before an underscore character is a special code for each volunteer. The second number after an underscore character refers to the order of the scent sampling.

Our olfactronic analysis showed successful individual separation/variance between different volunteers on the glass beads (see Figure 3). However, the system could not link the human scent extracted from the gun-fired cartridge cases to the volunteers. This could have been caused by the different kinds of background material that was provided by glass beads (see Figure 1) and cartridge cases (see Figure 2), despite the fact that data editing was done in an attempt to eliminate these differences. Another option could be that even if the significant substances for the identification withstand high temperatures, the explosion heat could slightly change them. Therefore, one of the solutions could be to equalize the conditions before the extraction by heating the glass beads to the same temperature to which the bullets are exposed when fired. Nevertheless, the surface differences from which the unknown trace was collected at the crime scene do not seem to play a significant role. This is important for further investigation, as the cartridge cases remaining at the crime scene could lay on many kinds of surfaces, and the analyses of the evidence must not depend on them. The inconsistency of the result from the previous experiment^4^ where the cartridge cases were successfully linked to the volunteer who had loaded the gun before the shooting could be based on the fact that another mass spectrometer (Pegasus BT-4D) with new commercial software (ChromaTOF, version 5.51.06.0) was used for this experiment. The software having issues with processing the chromatograms (mainly peak areas) may have contributed to the inconsistency.

As already stated in the Introduction, the fingerprints on cartridges found at a crime scene could be partially spoiled, and there are not enough minutiae for an unambiguous proper identification. However, both methods together, the scent analysis and fingerprints, could, in some cases, be strong evidence in court. The compatibility of both procedures would be complicated but not entirely impossible. The first step is to find the optimal order of the two methods. That means finding the method procedure of the first analysis that would not destroy the traces for the next one. The first option could be taking a high-resolution photograph of the fingerprint on the surface. That would be followed by scent extraction and further analysis. The issue is to use only visualization techniques that do not destroy the scent trace. In the reversed order of the two analyses, there is a question whether the fingerprints would remain on the surface even after the presented extraction of the scent.

For the second method, four double blind lineup procedures were performed with the four cartridge cases (the same ones used for the first olfactronics test). In every lineup, five different scent samples from five different volunteers were compared with the sniffing sample. The lineups were carried out by two handlers and four different dogs. The results showed that the canines can detect the human scent remaining on the cartridge case and successfully link it to the volunteer who loaded the gun. In two cases, the canine did not mark the volunteer on the first attempt, passing the lineup without marking any of the samples. This could be based on the fact that these samples were the ones that were exposed to the cartridge cases only for 30 min and 1 h, respectively. This allows a conclusion to be drawn that the time of exposure of the traces to the Aratex fabric should be longer. Based on the results, the canines were able to correctly identify the shooter in 67% of cases on the first attempt. They did not misidentify any of the deceptive scent samples. In the remaining 33% of cases, the dogs initially did not mark any samples but were successful in identifying the correct one in the next round, as previously noted.

## Conclusions

Two methods for comparing four scent traces from a simulated crime scene with 22 different volunteer scent samples were used. The aim was to find a certain volunteer who loaded the gun using eight analogous cartridges. Four samples of cartridge cases were then subjected to olfactronic analysis. These were collected from a heterogeneous crime scene. These four analogous samples were also compared by trained police canines. The olfactory method was successful and correctly identified the shooter. Both methods indicated that the surface (plastic sheet, wooden palette, and concrete floor) from which the scent traces were collected does not play a significant role in the identification. The results from the forensic olfactronic method showed the ability to distinguish each volunteer sampled on the glass beads; however, it has not yet been possible to link the fired cartridge cases to any of the volunteers. In the future, this problem can be eliminated by solving the problems with processing the peaks with commercial software that comes with the two-dimensional gas chromatograph. On the other hand, the issue could also be solved by equalizing the parameters on both surfaces (cartridge cases and glass beads) by heating the glass beads after the scent is collected on them. This heating would partly simulate the process of firing the gun.

Additionally, olfactronic identification could also be complemented by a dactyloscopy comparison as well. Since both can be crucial for the investigation and together can form a desirable chain of evidence. The only question is in which order should the methods be used for the optimal analysis of both.

The future prospects for the study include expanding the research to involve more volunteers and diversifying the types of weapons and ammunition tested. Additionally, further optimization of the sample preparation process is necessary, particularly to determine whether heating the glass beads to simulate gunfire can effectively link unknown samples to the shooter. Finally, addressing the issue of data alignment will be a critical step moving forward.
